# Engineering *Pseudomonas putida* for isoprenoid production by manipulating endogenous and shunt pathways supplying precursors

**DOI:** 10.1186/s12934-019-1204-z

**Published:** 2019-09-09

**Authors:** Sofía Hernandez-Arranz, Jordi Perez-Gil, Dominic Marshall-Sabey, Manuel Rodriguez-Concepcion

**Affiliations:** Centre for Research in Agricultural Genomics (CRAG), CSIC-IRTA-UAB-UB, Barcelona, Spain

**Keywords:** DXS, Isoprenoids, Lycopene, MEP pathway, *Pseudomonas putida*

## Abstract

**Background:**

The soil bacterium *Pseudomonas putida* is a promising platform for the production of industrially valuable natural compounds. In the case of isoprenoids, the availability of biosynthetic precursors is a major limiting factor. In *P. putida* and most other bacteria, these precursors are produced from pyruvate and glyceraldehyde 3-phosphate by the methylerythritol 4-phosphate (MEP) pathway, whereas other bacteria synthesize the same precursors from acetyl-CoA using the unrelated mevalonate (MVA) pathway.

**Results:**

Here we explored different strategies to increase the supply of isoprenoid precursors in *P. putida* cells using lycopene as a read-out. Because we were not aiming at producing high isoprenoid titers but were primarily interested in finding ways to enhance the metabolic flux to isoprenoids, we engineered the well-characterized *P. putida* strain KT2440 to produce low but detectable levels of lycopene under conditions in which MEP pathway steps were not saturated. Then, we compared lycopene production in cells expressing the *Myxococcus xanthus* MVA pathway genes or endogenous MEP pathway genes (*dxs*, *dxr*, *idi*) under the control of IPTG-induced and stress-regulated promoters. We also tested a shunt pathway producing isoprenoid precursors from ribulose 5-phosphate using a mutant version of the *Escherichia coli ribB* gene.

**Conclusions:**

The most successful combination led to a 50-fold increase in lycopene levels, indicating that *P. putida* can be successfully engineered to substantially increase the supply of metabolic substrates for the production of industrially valuable isoprenoids.

## Background

Isoprenoids (also known as terpenoids) constitute the largest class of natural products, with tens of thousands of different structures found in all kingdoms of life [1]. Many of them are high value compounds due to their diverse range of applications in areas such as human nutrition and pharmaceutical industry [2]. Most economically-relevant isoprenoids are typically found at low levels in their natural sources. This, together with the increasing demand for novel isoprenoid structures with new or improved bioactivities, has spurred many metabolic engineering and synthetic biology projects focused on boosting isoprenoid production. Much effort has been devoted towards their synthesis in microbial hosts, mostly employing *Escherichia coli* and *Saccharomyces cerevisiae*. However, interference with the endogenous cell metabolism and toxic effects associated with the production of most isoprenoids, among others, are important issues that often prevent reaching commercially relevant yields [3].

In this context, the stress-resistant bacterium *Pseudomonas putida* is a promising alternative platform for isoprenoid production and storage. Some *P. putida* strains can tolerate much higher level of isoprenoids than other microorganisms (e.g. its growth is not affected by concentrations of the monoterpene geranic acid that are toxic to *E. coli* or *S. cerevisiae* [4]). The high tolerance to otherwise toxic compounds of *P. putida* cells is in part due to fine-tuning of lipid fluidity to adjust membrane functions, activation of a general stress-response system, increased energy generation and induction of efflux pumps [5]. Additionally, *P. putida* is amenable to targeted genetic modifications and it has been targeted in biofactories design and biodegradation studies due to its robust metabolism, among others characteristics. Several industrial processes based on this bacterium are being exploited for the production of fine chemicals (e.g. 2-quinoxalinecarboxylic acid, 5-methylpirazine-2-carboxylic acid, chiral amines and 4-(hydroxypyridin-3-yl)-4-oxobutyrate), and the production of many different compounds, including isoprenoids, is under study [6]. *P. putida* has actually been used for the production of some isoprenoids using biotransformation approaches to obtain oxidation products of the plant monoterpene limonene [7] or de novo biosynthesis of the monoterpene geranic acid [4] or the carotenoids zeaxanthin and β-carotene [8–10]. Despite the low number of available studies, the achievement of substantial yields of isoprenoids such as geranic acid and zeaxanthin and the high tolerance of *P. putida* to some of these compounds, usually toxic for other microbial models, illustrate the high potential of this bacterium to become a platform of choice for the industrial production of valuable isoprenoids. An important conclusion of these studies is that enhancing the supply of isoprenoid precursors is crucial for success as the bacterium only produces minor amounts of such precursors [7].

**Fig. 1:**
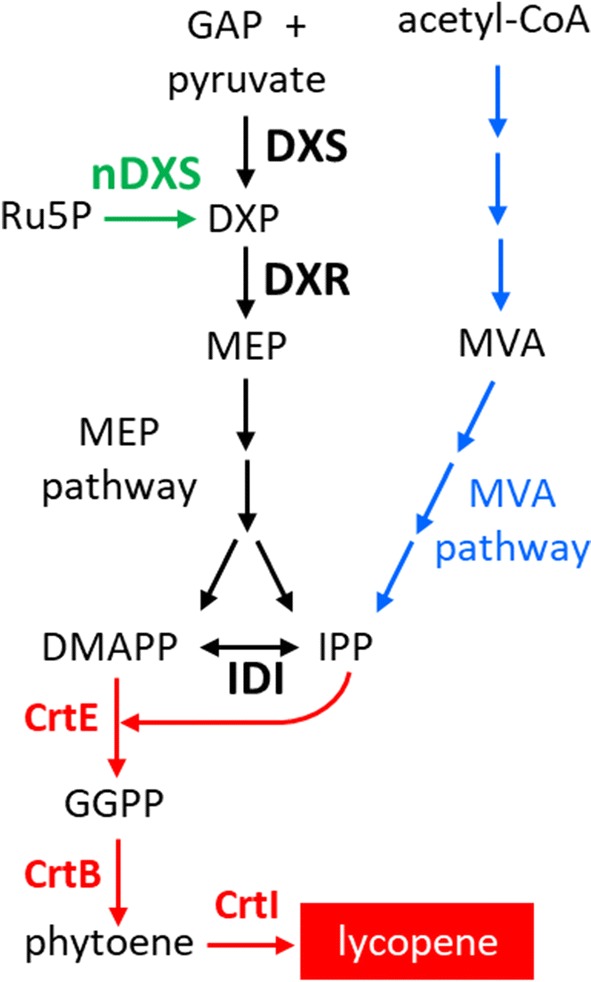
Pathways for isoprenoid synthesis. The *P. putida* endogenous MEP pathway is shown in black. Exogenous pathways added to *P. putida* in this work are shown in color: shunt pathway from *E. coli* in green, MVA pathway from *M. xanthus* in blue, and lycopene pathway from *P. ananatis* in red. Enzymes are marked in bold

All isoprenoids derive from two universal 5-carbon (C5) precursors: isopentenyl diphosphate (IPP) and its double-bond isomer dimethylallyl diphosphate (DMAPP). In nature there are two main pathways for IPP and DMAPP production: the mevalonate (MVA) pathway and the methylerythritol 4-phosphate (MEP) pathway (Fig. 1). As most bacteria, *P. putida* only harbors the MEP pathway. By contrast, other bacteria as well as archaea, fungi, and animal cells produce their isoprenoids from MVA-derived precursors [11]. Because IPP and DMAPP availability is limiting for isoprenoid biosynthesis, the MVA and MEP pathways have been the targets of many metabolic engineering efforts to increase the supply of isoprenoid precursors in host cells [3]. A virtually unexplored alternative, however, is the use of non-canonical pathways to synthesize such precursors. Bacteria show a remarkable plasticity for the production of their isoprenoid precursors. For example, the production of deoxyxylulose 5-phosphate (DXP) in the first and main rate-determining step of the MEP pathway can be achieved by different pathways [12]. The canonical enzyme, DXP synthase (DXS), produces C5 DXP from C3 pyruvate and glyceraldehyde 3-phosphate (GAP), but several mutant versions of the *E. coli ribB* gene were found to encode enzymes with a “new DXS” (or nDXS) activity that made DXP using alternative substrates (Fig. 1) [13, 14]. The wild-type *ribB* gene encodes 3,4-dihydroxy-2-butanone-4-phospate (DHBP) synthase, which converts C5 ribulose-5-phosphate (Ru5P) into formate and DHBP, the biosynthetic precursor of the xylene ring of riboflavin [15]. Ru5P appears to be the substrate that nDXS enzymes convert into DXP [13].

Here we have compared the performance of previously known and novel strategies to improve the supply of IPP and DMAPP for isoprenoid biosynthesis in KT2440, the best characterized *P. putida* strain. Using as read-out the production of lycopene, a red carotenoid pigment with great commercial and nutritional importance, we increased isoprenoid precursor levels by introducing an exogenous MVA pathway for IPP and DMAPP synthesis, by producing DXP via nDXS, and by expressing DXS and other MEP pathway enzymes under the control of different promoters.

## Results

### Pseudomonas putida KT2440 can be engineered for lycopene production

**Fig. 2:**
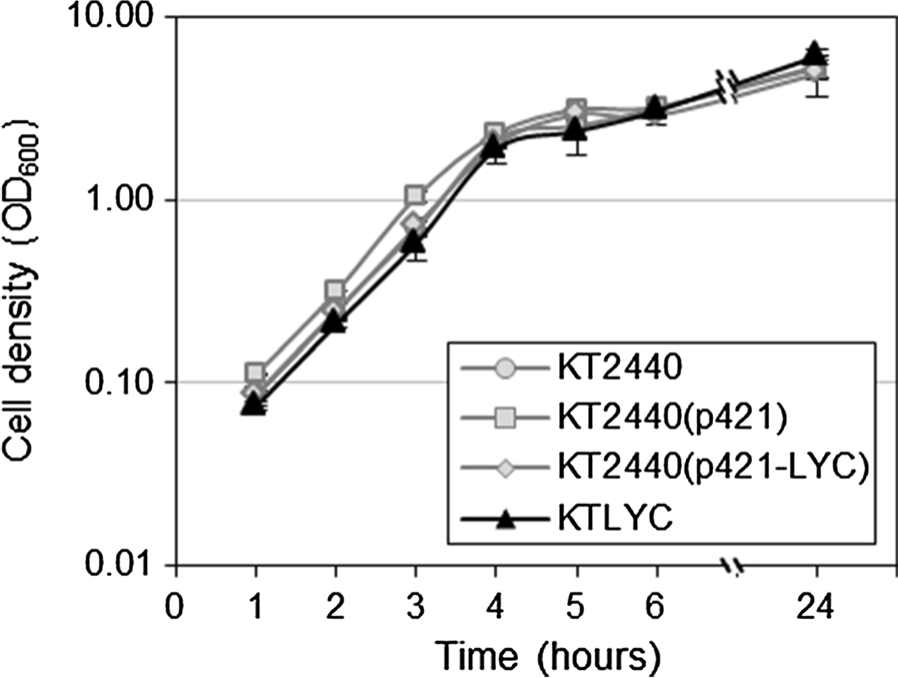
Cell density of *P. putida* strains producing lycopene. The indicated strains were grown in LB and their growth was measured as optical density at 600 nm (OD_600_). Values shown are the mean ± SD of three independent assays

To use lycopene as a read-out of isoprenoid precursor availability in *P. putida*, a lycopene-producing operon (LYC) containing the *crtE*, *crtB* and *crtI* genes from *Pantoea ananatis* [16], was expressed in the strain KT2440. *crtE* encodes the enzyme geranylgeranyl diphosphate (GGPP) synthase, which catalyzes the addition of three molecules of IPP to one DMAPP unit to yield GGPP, the immediate precursor for carotenoids. C20 GGPP is converted to C40 lycopene via phytoene by phytoene synthase and phytoene desaturase, encoded by *crtB* and *crtI* respectively (Fig. 1). Two strategies were used to introduce the LYC operon into *P. putida*. First, the operon containing the three genes and their own promoter was cloned into the vector pSEVA421 [17]. The plasmid obtained, p421-LYC, was introduced into the strain KT2440 and its ability to produce lycopene was compared to that of cells carrying the empty vector. After 24 h growing in LB, cells were harvested for lycopene extraction and quantification by HPLC. While no lycopene was detected in empty vector control cells, measurable levels of this red carotenoid (1.223 ng/ml of culture) were found in the strain expressing the LYC operon (Table 1). Cell growth estimated as optical density at 600 nm (OD_600_) was not affected in any of the strains (Fig. 2).

**Table 1:** Cell density (turbidity at 600 nm) and lycopene accumulation (measured by HPLC) in cells cultured during 24 h in LB

Strain	Cell density (OD_600nm_)	Lycopene accumulation (ng/ml culture)
KT2440	5.417 ± 0.791	0.000 ± 0.000
KT2440(p421)	5.293 ± 0.623	0.000 ± 0.000
KT2440(p421-LYC)	4.930 ± 1.191	1.223 ± 0.185
KTLYC	6.157 ± 0.551	0.106 ± 0.026

Values shown are the mean ± SD for three independent assays

Once we confirmed the ability for lycopene synthesis of *P. putida* cells, the *crtE*–*crtB*–*crtI* operon with its own promoter was inserted into the Tn7 *att* site in the chromosome of KT2440, using a Tn7-based delivery system [18], to yield the strain KTLYC. Strains KT2440 and KTLYC were grown for 24 h in LB and lycopene accumulation was then measured by HPLC. Growth of both strains was basically identical (Fig. 2a) whereas, as expected, lycopene was only detected in KTLYC (Table 1). However, the amounts produced in this strain (0.106 ± 0.026 ng/ml culture) were an order of magnitude lower than those found in KT2440 cells containing the plasmid p421-LYC (Table 1), perhaps due to differences in copy number or DNA context (genomic vs. plasmidial). In any case, these results allowed to conclude that, under our experimental conditions, the intermediate steps of the endogenous MEP pathway are not saturated in strain KTLYC. We therefore selected this strain for exploring ways of increasing the supply of MEP-derived isoprenoid precursors (IPP and DMAPP) in *P. putida* cells.

### Lycopene production in P. putida KTLYC is enhanced by expressing an exogenous MVA pathway

**Fig. 3:**
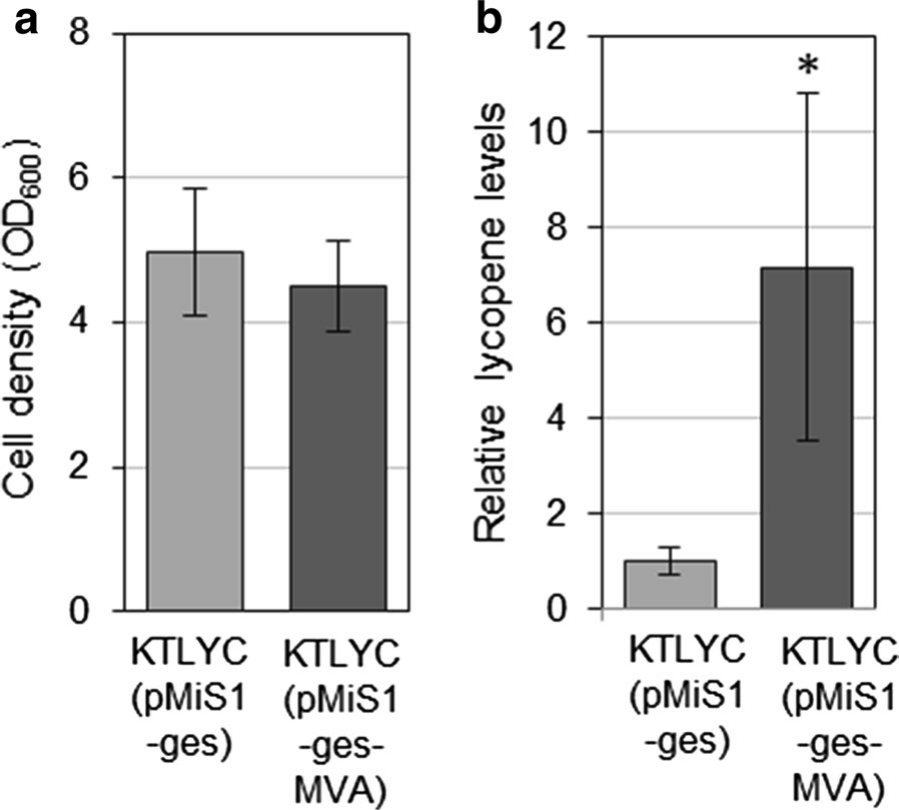
Lycopene production in *P. putida* KTLYC cells harboring the MVA pathway. KTLYC cells transformed with vectors either containing or not the MVA pathway were cultured in LB. Expression of the MVA pathway operon was induced 3 h after inoculation by adding 0.2% (w/v) *L*-rhamnose to the culture. After 24 h, cell growth was estimated as optical density at 600 nm (OD_600_) and lycopene accumulation was calculated by measuring the absorbance at 472 nm of acetone extracts. **a** OD_600_ values. **b** Lycopene levels normalized to cell density and represented relative to those in cells without the MVA pathway. Values shown are the mean ± SD of three independent assays. The asterisk indicates statistically significant difference (*T* test, *P*-value < 0.05)

As a first strategy to enhance the supply of metabolic precursors for isoprenoid biosynthesis in *P. putida*, we introduced an exogenous MVA pathway (Fig. 1) in the KTLYC strain. The vector pMiS1-ges-MVA, containing the bacterium *Myxococcus xanthus* genes required to convert acetyl-CoA into IPP and DMAPP under the control of the *rhaP*_*BAD*_ promoter [4], was introduced into *P. putida* KTLYC. As control, the plasmid pMiS1-ges (similar to pMiS1-ges-MVA but without the MVA pathway genes) was also introduced into KTLYC. Cells were cultured in LB medium and at the mid-exponential phase, expression of plasmid vector genes was induced by *L*-rhamnose. After 24 h, cells were harvested for lycopene extraction and quantification. Lycopene levels were estimated by measuring absorbance at 472 nm, normalized to cell density (OD_600_), and represented relative to controls (Fig. 3). Lycopene production was about sevenfold higher in the strain harboring the MVA pathway without this having an impact in cell growth (Fig. 3). These results show that promoting the supply of IPP and DMAPP from acetyl-CoA by using an exogenous MVA pathway can efficiently improve lycopene production.

### Up-regulation of endogenous DXS greatly improves isoprenoid precursor supply in KTLYC cells

**Fig. 4:**
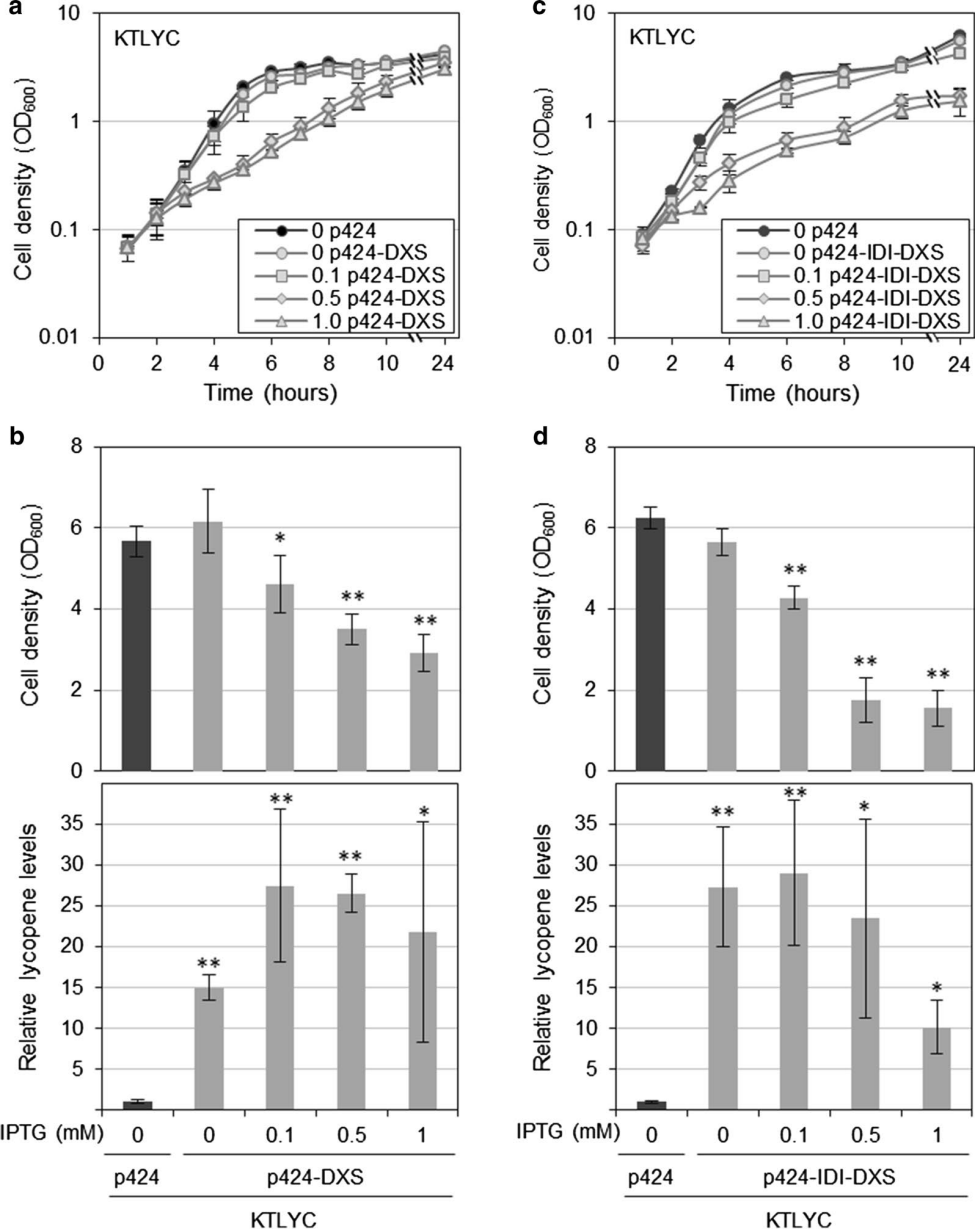
Cell density and lycopene content of *P. putida* KTLYC cells overproducing DXS. Overnight cultures of KTLYC cells transformed with the indicated vectors were inoculated in LB. Expression of the cloned genes was induced 1 h after inoculation by adding the indicated amounts of IPTG (mM) to the culture. After 24 h, growth was estimated from optical density at 600 nm (OD_600_) and lycopene accumulation was calculated by measuring the absorbance at 472 nm of acetone extracts. **a** Cell density kinetics of KTLYC cells transformed with either p424-DXS or empty pSEVA424 plasmids, estimated from OD_600_ values. **b** Cell density and lycopene content of the strains shown in **a** at 24 h. **c** Cell density kinetics of KTLYC cells transformed with either p424-IDI-DXS or empty pSEVA424 plasmids, estimated from OD_600_ values. **d** Cell density and lycopene content of the strains shown in **c** at 24 h. Values shown are the mean ± SD of three independent assays. Asterisks indicate statistically significant differences (T-test, *P*-value < 0.05 * or 0.01 **) relative to KTLYC(p424) controls grown without IPTG

**Fig. 5:**
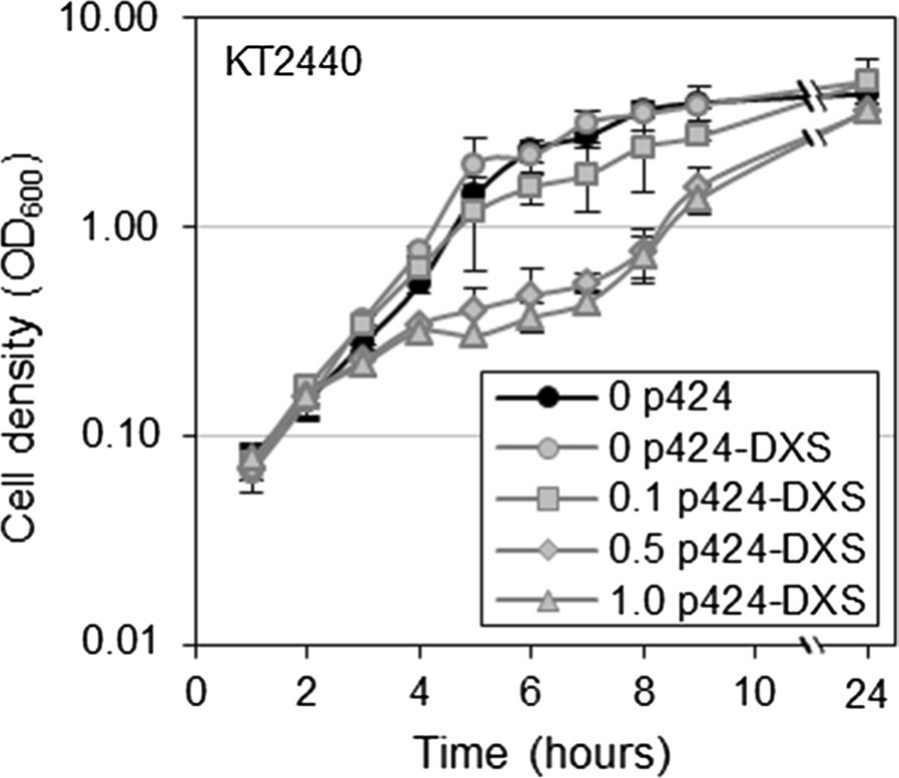
Effect of DXS overproduction on KT2440 cell growth. *P. putida* KT2440 cells harbouring the vector p424-DXS or the empty pSEVA424 plasmid were inoculated in LB and, after 1 h, the medium was supplemented with the indicated concentration of IPTG (mM). Increase in cell growth was measured as optical density at 600 nm (OD_600_). Values shown are the mean ± SD of three independent assays

Up-regulation of the levels of several MEP pathway enzymes has been reported to improve flux through the pathway in bacteria, with the clearest effect typically observed with DXS when overexpressed at moderate levels. To test whether DXS was also a rate-limiting enzyme in *P. putida*, the *dxs* gene was amplified from KT2440 cells and cloned into the pSEVA424 plasmid [17] under the transcriptional control of the strong and IPTG-inducible *Ptrc* promoter. After transformation, KTLYC cells harboring either the generated vector (p424-DXS) or an empty plasmid were cultured in LB and 1 h after inoculation the medium was supplemented with different concentrations of IPTG. While 0.1 mM IPTG had no impact on culture growth, higher concentrations of the inducer (e.g. 0.5 mM and 1 mM) caused a much slower growth (Fig. 4a). Lycopene accumulation in KTLYC(p424) and KTLYC(p424-DXS) cells was quantified after 24 h by measuring absorbance at 472 nm (Fig. 4b). The presence of the plasmid p424-DXS caused a dramatic (15-fold) increase in lycopene accumulation even in the absence of IPTG (Fig. 4b). The positive effect of DXS on lycopene production was even stronger when *dxs* gene expression was induced with IPTG (25-fold). The observation that KT2440 cells lacking the capacity to produce lycopene showed very similar growth curves when transformed with p424-DXS and incubated with IPTG (Fig. 5) suggested that the growth phenotype is not caused by the accumulation of toxic levels of lycopene but results from the overexpression of the *dxs* gene.

GGPP required for lycopene production is formed by adding three IPP molecules to one DMAPP acceptor, whereas bacterial isoprenoids are most often derived from farnesyl diphosphate (FPP), that is formed from two IPP and one DMAPP [11]. Because an unbalanced production of isoprenoid precursors has been shown to be detrimental for bacterial growth [19], we next investigated whether enhancing the interconversion of IPP and DMAPP might positively impact cell growth and lycopene accumulation in strains overexpressing DXS. The IPP/DMAPP ratio is regulated by the enzyme isopentenyl diphosphate isomerase (IDI) that interconverts IPP and DMAPP molecules (Fig. 1). The *idi* gene from *E. coli* was cloned into the p424-DXS vector and the resulting p424-IDI-DXS plasmid was introduced into the strain KTLYC. Positive transformants were grown with or without IPTG as described above (Fig. 4c) and both cell growth and lycopene accumulation were recorded at 24 h (Fig. 4d). In the absence of IPTG, lycopene production tended to be slightly higher in cells harboring p424-IDI-DXS compared to p424-DXS, but the difference was not statistically significant. Induction with increasing concentrations of IPTG did not improve cell growth or lycopene production on cells harboring p424-IDI-DXS (Fig. 4d) compared to p424-DXS (Fig. 4b). On the contrary, growth of KTLYC(p424-IDI-DXS) cells was reduced compared to that of IDI-lacking strains at IPTG concentrations higher than 0.1 mM. Addition of 1 mM IPTG to the medium also reduced the capacity of the cells for lycopene accumulation (Fig. 4). In summary, IPP/DMAPP imbalance might influence lycopene biosynthesis but it appears not to be a major factor limiting lycopene yields in *P. putida* cells. While DXS activity is much more relevant to regulate flux to isoprenoid biosynthesis, a fine control of its levels is critical to prevent deleterious effect on cell growth.

### Use of the stress-regulated promoter PkatA to express IDI and DXS has a modest effect on lycopene production

**Fig. 6:**
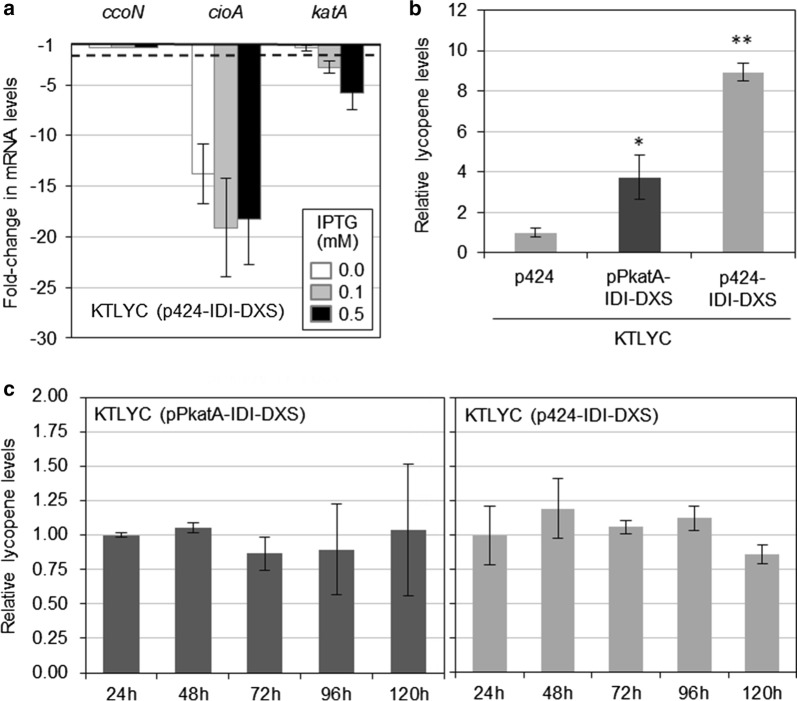
Test of the *P. putida PkatA* promoter. **a** Transcript levels of *ccoN*, *cioA* and *katA* in KTLYC(p424-IDI-DXS) cells grown in the presence of the indicated concentrations of IPTG. The graph represents the ratio of the mRNA levels in KTLYC(p424-IDI-DXS) cells relative to KTLYC(p424) controls grown under the same conditions. Error bars indicate SD. **b** Lycopene accumulation in the indicated strains grown for 24 h in LB without IPTG. Values shown are the mean ± SD of three independent assays. Asterisks indicate statistically significant differences (T-test, *P*-value < 0.05 * or 0.01 **) relative to KTLYC(p424) controls. **c** Lycopene accumulation in the indicated strains grown in LB without IPTG for the indicated times (in hours). Values are shown relative to 24 h samples and represent the mean ± SD of three replicates in two independent assays

To overcome the toxic effects associated with a too strong DXS activity, we next looked for alternative promoters that could be down-regulated when cell growth is compromised. As starting point, we studied the expression of the stress-regulated genes *ccoN* (PP4250), *cioA* (PP4651) and *katA* (PP0481) in KTLYC(p424-IDI-DXS) and KTLYC(p424) cells exposed to different concentrations of IPTG (Fig. 6a). The operons *ccoN1O1Q1P1* and *cioAB* encode two terminal oxidases, the cytochrome cbb3-1 oxidase and the cyanide-insensitive oxidase (CIO), respectively, and they are up-regulated under low-oxygen conditions [20]. The expression of the *katA* gene, encoding one of the major peroxide-degrading enzyme directly eliminating oxidants in the cell, is regulated by matric and oxidative stress [21]. The levels of *ccoN*, *cioA* and *katA* transcripts in the two strains under different growth conditions were estimated by real time quantitative RT-PCR and represented as relative fold-changes in KTLYC(p424-IDI-DXS) compared to KTLYC(p424) cells (Fig. 6a), No changes were observed in the case of *ccoN* whereas *cioA* mRNA levels were drastically reduced in KTLYC(p424-IDI-DXS) cells independent of IPTG concentration (i.e. regardless of DXS levels). In the case of *katA*, similar transcript levels were found in the two strains when growing without the inductor but were reduced in KTLYC(p424-IDI-DXS) when IPTG was added to the medium to induce *idi*-*dxs* co-transcription (Fig. 6a). The reduction was moderate with 0.1 mM IPTG, a concentration that hardly impacts growth of this strain (Fig. 4c). When growth was inhibited at 0.5 mM IPTG, however, a clear sixfold reduction in *katA* expression was detected (Fig. 6a). We therefore concluded that the *PkatA* promoter might be repressed when DXS (and IDI) activities are high enough to cause growth-repressing effects.

With the aim of adjusting enhanced isoprenoid precursor supply to actual cell metabolism and growth, we next used the *PkatA* promoter [22] to construct a synthetic *PkatA*-*idi*-*dxs* operon. KTLYC strains harboring the plasmids pPkatA-IDI-DXS or p424-IDI-DXS, as well as the empty pSEVA424 vector, were cultured in LB without IPTG and lycopene accumulation levels were analyzed every 24 h during 5 days. After the first 24 h (Fig. 6b) KTLYC(pPkatA-IDI-DXS) cells showed fourfold higher levels of lycopene than KTLYC(p424) but half as much compared to KTLYC(p424-IDI-DXS). Neither KTLYC(pPkatA-IDI-DXS) nor KTLYC(p424-IDI-DXS) cells produced more lycopene at longer incubation times (Fig. 6c). It is therefore possible that the *Ptrc* promoter present in the p424-IDI-DXS construct leads to higher enzyme levels than the *PkatA* promoter even without the IPTG inducer, hence being more efficient to increase the MEP pathway flux.

### Depletion of pyruvate and GAP might contribute to the cell growth defects associated with DXS overexpression

**Fig. 7:**
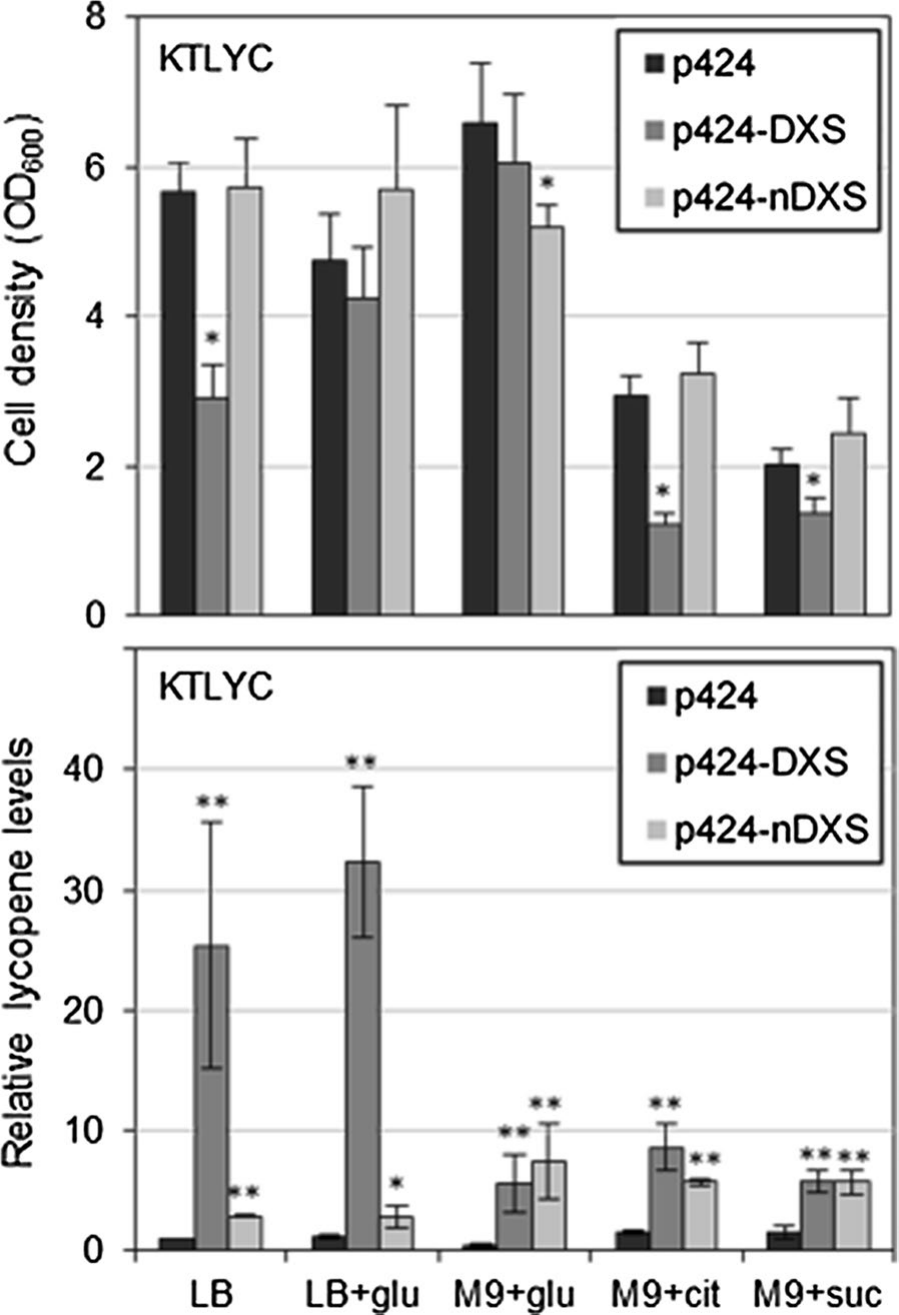
Growth and lycopene levels of KTLYC cells producing either DXS or nDXS. The strains KTLYC(p424), KTLYC(p424-DXS) and KLYC(p424-nDXS) were cultured in the indicated mediums: LB, LB supplemented with 50 mM glucose, or M9 minimal salt medium containing 50 mM glucose, 30 mM citrate or 30 mM succinate as carbon source. Expression of genes encoding DXS or nDXS was induced with 1 mM IPTG 1 h after inoculation. After 24 h, growth was estimated as optical density at 600 nm (OD_600_) (upper graph) and lycopene accumulation was calculated by measuring the absorbance at 472 nm of acetone extracts of the cells and normalized to cell density (lower graph). Values shown are the mean ± SD of three independent assays. Asterisks indicate statistically significant differences (T-test, *P*-value < 0.05 * or 0.01 **) relative to KTLYC(p424) controls

When KT2440 cells are grown in the presence of glucose, they run a biochemical cycle that merges activities belonging to the Entner–Doudoroff (ED) pathway, the Embden–Meyerhof–Parnas (EMP) pathway and the pentose phosphate pathway that produces equimolar amounts of pyruvate and GAP, favors NADPH formation, and helps the bacteria to cope from different types of environmental stress [6, 23]. To test whether the defective growth phenotype observed in DXS-overproducing *P. putida* cells might result from metabolic imbalance caused by low pyruvate and GAP levels, we grew KTLYC(p424-DXS) and KTLYC(p424) cells in LB either supplemented or not with 50 mM glucose. IPTG was added at high concentration (1 mM) at the beginning of the exponential phase and cell growth and lycopene accumulation were analyzed after 24 h. As shown in Fig. 7, the sharp difference in cell growth between KTLYC(p424-DXS) and KTLYC(p424) strains was completely abolished in glucose-supplemented medium. Glucose, but not other carbon sources such as citrate or succinate, also rescued growth defects observed when cells were grown in minimal M9 medium (Fig. 7). These results support the conclusion that depletion of GAP and pyruvate when DXS levels are too high is a major cause for the growth defects observed in engineered *P. putida* cells. Furthermore, the addition of glucose to LB medium increased lycopene accumulation per cell (Fig. 7), suggesting that IPP and DMAPP supply for isoprenoid biosynthesis could be improved by increasing the availability of both GAP and pyruvate or by finding alternative sources for DXP synthesis.

**Fig. 8:**
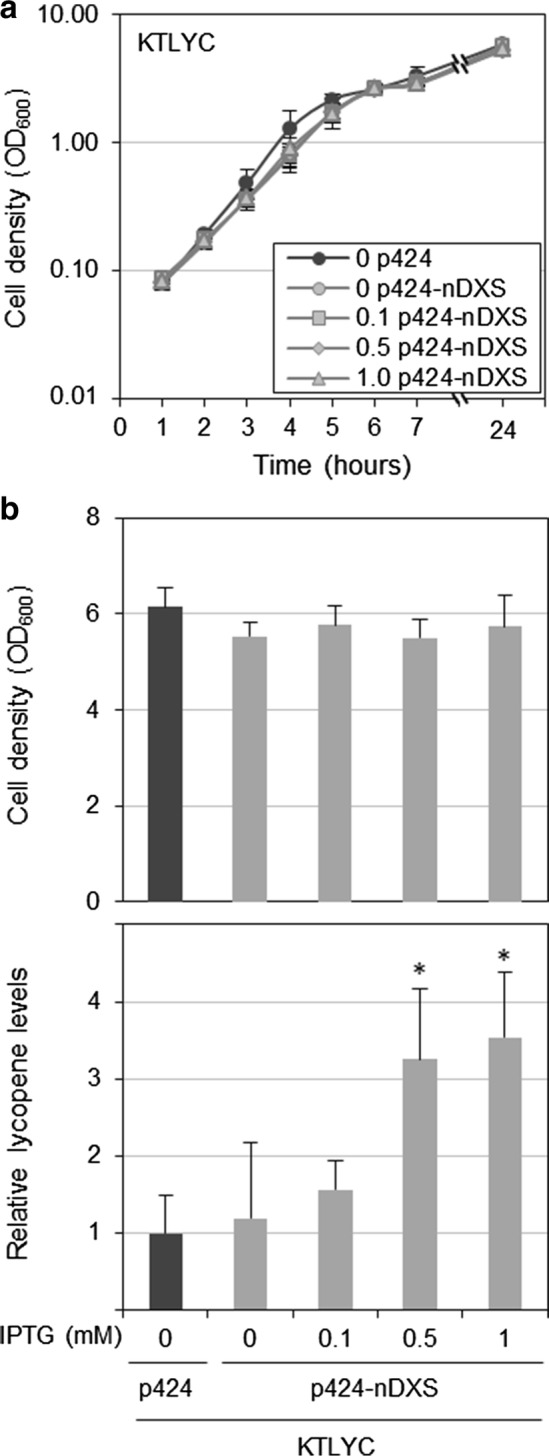
Growth and lycopene content of *P. putida* KTLYC cells overproducing nDXS. KTLYC cells transformed with the indicated vectors were cultured in LB. Expression of the cloned genes was induced 1 h after inoculation by adding the indicated amounts of IPTG (mM) to the culture. After 24 h, growth was measured as optical density at 600 nm (OD_600_) and lycopene accumulation was calculated by measuring the absorbance at 472 nm of acetone extracts. **a** Cell density kinetics of KTLYC cells transformed with either p424-nDXS or empty pSEVA-424 plasmids estimated from OD_600_ values. **b** Cell density and lycopene content of the strains shown in **a** at 24 h. Values shown are the mean ± SD of three independent assays. Asterisks indicate statistically significant differences (T-test, *P*-value < 0.05) relative to KTLYC(p424) controls grown without IPTG

It was previously reported that single mutations in the *E. coli ribB* gene, encoding DHBP synthase, allow the mutant enzymes (nDXS) to produce DXP from Ru5P and hence complement the absence of the DXS enzyme in *E. coli* [13, 14]. Because DXP production by nDXS enzymes does not directly consume pyruvate and GAP and it is less efficient compared to DXS, we reasoned that using nDXS enzymes might overcome some of the problems associated with DXS overexpression. Although several mutations in *ribB* allow DXP synthesis, for this work we used the nDXS version harboring a missense mutation resulting in a G108S change [13, 14]. Following a similar strategy to that described for DXS, we generated the plasmid p424-nDXS and used it to transform KTLYC cells. The resulting strain and the KTLYC(p424) control were cultured in LB in the absence or presence of IPTG (added 1 h after inoculation) and lycopene accumulation was analyzed after 24 h. Unlike that observed for KTLYC(p424-DXS) and KTLYC(p424-IDI-DXS) strains (Fig. 4), growth of KTLYC(p424-nDXS) cells was not affected by IPTG at any of the concentrations tested (Fig. 8a). Also different to DXS-producing strains, KTLYC cells producing nDXS showed a good correlation between IPTG and lycopene levels (Fig. 8b). However, lycopene accumulation in KTLYC(p424-nDXS) cells at the highest IPTG concentration tested (1 mM) was only threefold higher than that reached in the absence of inducer (Fig. 8b).

Ru5P, the substrate of nDXS, is an intermediate of the pentose phosphate pathway, which is not very active in *P. putida* [6, 23]. In complete (LB) medium, the addition of 50 mM glucose had no effect on lycopene production when KTLYC(p424-nDXS) cells were induced with 1 mM IPTG (Fig. 7). In minimal (M9) medium, similar levels of lycopene were produced with glucose, citrate, or succinate. It is interesting, however, that whereas lycopene accumulation in KTLYC(p424-DXS) cells was much lower in M9 than in LB medium, KTLYC(p424-nDXS) cells showed similar lycopene levels in both media (Fig. 7). We therefore conclude that the main factor limiting isoprenoid production in the KTLYC(p424-nDXS) strain is not Ru5P availability but the capacity of nDXS to synthesize DXP.

### Combination of DXS and DXR further increases lycopene accumulation

**Fig. 9:**
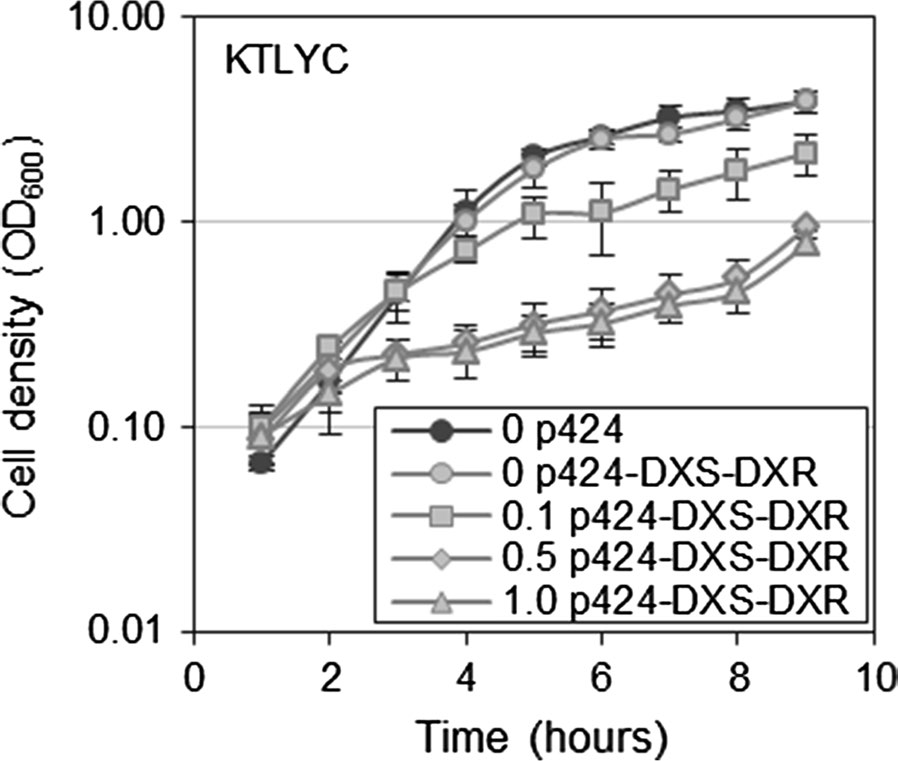
Cell growth of *P. putida* KTLYC strains overproducing DXS and DXR. Overnight cultures of KTLYC cells transformed with the indicated plasmids were inoculated in LB and, after 1 h, the indicated concentration of IPTG (mM) was added to the medium. Graph shows the increase in cell density at 600 nm (OD_600_). Values shown are the mean ± SD of three independent assays

**Fig. 10:**
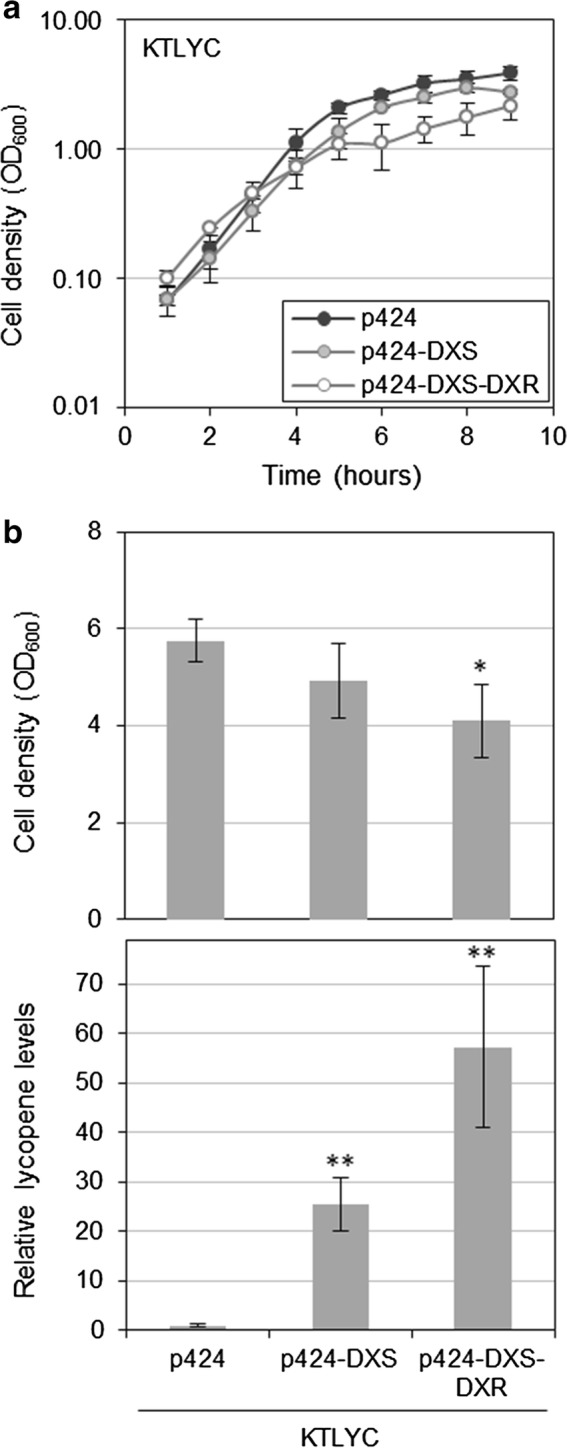
Cell growth and lycopene accumulation in *P. putida* KTLYC cells overproducing DXS and DXR. Overnight cultures of KTLYC cells transformed with the indicated vectors were inoculated in LB and induced to express the cloned genes with 0.1 mM IPTG added 1 h after inoculation. **a** Cell density kinetics of the indicated strains estimated from OD_600_ values. **b** Cell density and lycopene content of the strains shown in **a** at 24 h. Values shown are the mean ± SD of three independent assays. Asterisks indicate statistically significant differences (T-test, *P*-value < 0.05 * or 0.01 **) relative to KTLYC(p424) controls

As a last strategy to up-regulate the endogenous MEP pathway and increase lycopene synthesis in *P. putida* KTLYC, the endogenous enzyme DXP reductoisomerase (DXR) was overproduced in this strain either alone or together with DXS. DXR catalyzes the second step of the MEP pathway, i.e. the reduction of DXP to MEP (Fig. 1). After generating construct p424-DXS-DXR and transforming KTLYC cells, growth of the generated strain in LB medium was monitored upon adding IPTG 1 h after inoculation (Fig. 9). In agreement with our conclusion that cell growth defects observed when the MEP pathway flux is up-regulated are mainly due to depletion of pyruvate and GAP, growth of KTLYC(p424-DXS-DXR) cells was compromised even at low concentrations of IPTG such as 0.1 mM (Fig. 9), which had no impact on the growth of KTLYC(p424-DXS) cells (Fig. 4a). To test whether the expected up-regulation of the MEP pathway flux in KTLYC(p424-DXS-DXR) cells had a positive impact on lycopene accumulation, we next compared the levels of this carotenoid in KTLYC cells transformed with p424-DXS-DXR, p424-DXS, or with an empty vector and treated with 0.1 mM IPTG 1 h after inoculation. After 24 h, cells harboring the p424-DXS-DXR construct accumulated 50-fold higher lycopene levels per cell that KTLYC(p424) control. When compared to cells overexpressing only one of the two MEP pathway genes, KTLYC(p424-DXS-DXR) cells produced twice as much lycopene as KTLYC(p424-DXS) (Fig. 10).

## Discussion

Improving the supply of IPP and DMAPP for the biotechnological production of value-added isoprenoids in microorganisms has been the target of many studies. However, many other factors need to be taken into account to achieve an efficient production of isoprenoids without affecting the fitness of the cell. They include (A) depletion of metabolic substrates, (B) toxicity of accumulated intermediates or end products, and (C) saturation of the cell storage capacity. Strategies to deal with the last two problems are abundant, but less attention has been paid to the first, despite the negative growth impact of diverting the pools of central metabolites such as pyruvate and GAP to the synthesis of IPP and DMAPP. While most studies aimed to improve IPP and DMAPP supply have been done with well-characterized organisms such as *E. coli* (a model for bacteria that use the MEP pathway) and *S. cerevisiae* (a model for yeasts, which only use the MVA pathway), other microorganisms that show additional advantages for the production and accumulation of high isoprenoid titers remain scarcely explored. This is the case with *P. putida*, a bacterium that shows a very high tolerance to toxic compounds such as monoterpenes [4, 5]. An additional advantage of *P. putida* is that glucose is metabolized to produce balanced amounts of the MEP pathway substrates pyruvate and GAP [6, 10, 23]. These central metabolic substrates are produced in non-equivalent amounts in other bacteria such as *E. coli*, where this imbalanced availability of pyruvate and GAP can limit the activity of the MEP pathway [24]. Increasing the levels of both pyruvate and GAP in *P. putida* actually led to an increase (1.3-fold) in carotenoid (β-carotene) production [10], suggesting that the supply of metabolic substrates limits the MEP pathway flux. Our data reported here further support the conclusion that ensuring a proper supply of pyruvate and GAP is also important to upregulate isoprenoid production while preventing major interferences with overall microbial metabolism leading to defective growth.

To investigate different ways of stimulating the metabolic flux through the endogenous MEP pathway to isoprenoid precursors, we generated a *P. putida* strain (KTLYC) with an exogenous 3-gene operon to produce lycopene as a read-out. By transforming this strain with plasmids to either overexpress endogenous MEP pathway genes or incorporate alternative shunt pathways, we could multiply lycopene production (i.e. IPP and DMAPP supply) by 3 (with nDXS; Fig. 8), 7 (with the MVA pathway; Fig. 3), 25 (with DXS; Fig. 4) and 50 (with DXS and DXR; Fig. 10). It is most likely, however, that further optimization of at least some of these strategies or a combination of several could result in even higher increases. Additive and synergistic effects could also be achieved by improving the supply of MEP pathway substrates. A recent example is the rewiring of the *P. putida* core metabolism for glucose assimilation from the native Entner–Doudoroff to the linear Embden–Meyerhof–Parnas glycolysis, which improved the production of both pyruvate and GAP [10]. The metabolic plasticity shown by *P. putida*, which is able to metabolize a broad variety of carbon sources including fatty acids, glycerol and aromatic compounds [25], provides an additional opportunity to redesign the pools of substrates required for the synthesis of IPP and DMAPP. Feeding the bacteria with different carbon sources might help accumulate higher pools of pyruvate, GAP (i.e. glucose) or acetyl-CoA (i.e. fatty acids) and the combination of different carbon sources could help release the competition of the productive (exogenous) pathways with the housekeeping (endogenous) metabolism. The hierarchical utilization of different carbon sources [26] might limit our ability to combine fully active metabolic pathways to simultaneously feed productive and housekeeping pathways in the cell. However, our knowledge of the mechanisms controlling this hierarchy are constantly improving [27, 28], providing new engineering opportunities.

Among the novel strategies tested here, the use of nDXS to produce DXP from Ru5P was most successful as it improved lycopene accumulation up to threefold without negatively affecting cell growth (Fig. 8). While this increase is still far from that achieved with the canonical DXS enzyme (Fig. 4), it is important to keep in mind that nDXS was identified as a mutant enzyme which acquired the ability to produce DXP as a result of a point mutation and it is not optimized for DXP synthesis. Our estimation is that only about 10% of the Ru5P substrate is transformed into DXP by nDXS. Increasing the DXS activity of the mutant enzyme should be feasible and even fast if using the new automatized platforms available for adaptive laboratory evolution approaches [29, 30]. An optimized nDXS would allow a complementary strategy using the canonical DXS to push the MEP pathway using pyruvate and GAP up to the limits that show no effect on growth with an additional DXP input obtained from the transformation of Ru5P by nDXS. The availability of the crystal structure of the wild-type ribB protein [31] should allow to infer how the newly selected mutations could affect the DXS activity of nDXS, an information that could be very valuable to eventually create synthetic enzymes able to produce DXP from Ru5P or from substrates that do not interfere with the endogenous MEP pathway or other essential metabolic processes in the bacteria. As a complementary strategy, the supply of Ru5P might be enhanced by rewiring the pentose phosphate pathway. Ru5P is a C5 substrate and hence it is converted into DXP by nDXS without losing carbon (unless the reaction catalyzed by DXS, which starts with two C3 precursors and involves a decarboxylation). Together, the use of optimized versions of nDXS either alone or in combination with other strategies might increase IPP and DMAPP production to new high levels.

Another novel strategy that we tested was the fine regulation of DXS expression by using an endogenous promoter that responded to cell growth stress (Fig. 6). Using the *PkatA* promoter we only achieved a fourfold increase in lycopene accumulation compared to empty plasmid controls, suggesting that the transcriptional activity of this promoter in the conditions tested is too low to effectively boost the MEP pathway and hence to reach the stress conditions that could eventually repress transcription. It is likely that the use of stronger but still stress-responsive promoters might work better. The *PkatA* promoter might be engineered to increase its transcriptional activity while preserving its regulatory traits. Alternatively, analysis of the transcriptome of KTLYC(p424-DXS) cells grown in the presence of different concentrations of IPTG should allow to identify genes that are highly expressed when cells grow normally but are repressed when cell growth is compromised in an IPTG-dependent fashion (i.e. when DXS activity is too high). The promoters of such genes might be then used in synthetic operons to increase the supply of DXS (and IDI, DXR, nDXS, etc.) and hence the availability of isoprenoid precursors under optimal growth conditions but down-regulate them when growth-repressing effects appear. This will give time to the cells to readjust their metabolism and retake isoprenoid overproduction afterwards.

The more “classical” approaches that we tested, i.e. the use of an exogenous MVA pathway to produce IPP and DMAPP from acetyl-coA and the overexpression of MEP pathway genes, led us to conclude that we cannot directly extrapolate the results obtained with one bacterium (e.g. *E. coli*) to engineer another bacterium (e.g. *P. putida*). For example, using the MVA pathway typically works better than DXS up-regulation in *E. coli* [32–34] but we observed the opposite behavior in *P. putida*. A similar observation was previously reported in cyanobacteria growing in photoautotrophic conditions to produce isoprene [35]. The presence of the bacterial MVA pathway operon improved lycopene accumulation by sevenfold (Fig. 3), similar to the tenfold increase reported to cause the same operon on monoterpene production in *P. putida* DSM 12264 cells [4]. By contrast, the presence of a plasmid containing DXS was able to promote lycopene accumulation in KTLYC cells by 15-fold in the absence of any induction (Fig. 4b). The almost complete lack of effect of IDI overproduction on lycopene synthesis when the limiting role of DXS is released in *P. putida* (Fig. 4d) is also different to that observed in *E. coli* [29].

## Conclusion

Production of isoprenoids has been extensively engineered in classical microbial chassis such as *E. coli* and *S. cerevisiae* but much less effort has been made in *P. putida* despite the unique characteristics that make this organism a very attractive host for the industrial production of these metabolites. The present work analyzed different ways of enhancing the supply of isoprenoid precursors (IPP and DMAPP) in *P putida* strain KT2440 using lycopene as read-out. The most successful combination led to a 50-fold increase of lycopene accumulation. Under our experimental conditions, the production of limonene in *P. putida* cells engineered to synthesize this monoterpene was also boosted by the same MEP pathway constructs that improved lycopene levels, supporting the conclusion that the increases we observed in downstream isoprenoid products are indicative of a major up-regulation of the metabolic flux to IPP and DMAPP. Our work therefore represents an initial but important step towards engineering *P. putida* cells to produce high isoprenoid titers. The unique metabolic plasticity of *P. putida* together with its astonishing capacity to tolerate high levels of toxic isoprenoids such as monoterpenes and its amenability to genetic engineering make this bacterium a promising candidate to become a favorite microbial cell factory to produce high-value isoprenoids.

## Methods

### Bacterial strains and culture media

*Escherichia coli and P. putida* cells were cultured at 37 °C and 30 °C, respectively, with strong aeration. LB broth (10 g/l tryptone; 5 g/l yeast extract, 10 g/l NaCl) was used as complete growth medium. M9 minimal salts medium [36] was supplemented with trace elements [37], and either 30 mM citrate, 30 mM succinate or 50 mM glucose as the carbon source. When needed, antibiotic were added at the following concentrations: kanamycin 25 μg/ml, gentamicin 25 μg/ml, streptomycin 50 μg/ml (or 500 μg/ml when the host strain harboured a gentamicin-resistance determinant), ampicillin 100 μg/m. Cell density was monitored by measuring turbidity (optical density) at 600 nm (OD_600_).

To generate strain KTLYC, a 3788 bp DNA fragment containing the *P. ananatis* genes *crtE*, *crtB* and *crtI* and their own promoter was PCR-amplified from the vector pACCRT-EIB [16] and cloned into the *Not*I site of the Tn7-based delivery vector pTn7-M [18]. The generated plasmid, named pTn7-M-LYC, was transferred to *P. putida* strain KT2440 by tetra-partite mating using *E. coli* cc118λ*pir* (pTnS1) as the supplier of Tn7 transposase and *E. coli* HB101 (pRK600) as the transfer function donor, as previously described [18]. The correct insertion of the transposon at the *att* Tn7 site of the *P. putida* genome was checked by sequencing.

### Plasmids

Plasmid p421-LYC contains the *crtE*, *crtB* and *crtI* genes and their own promoter from *P. ananatis*. To generate this vector, the same 3788 bp DNA fragment inserted in the genome of the strain KTLYC was cloned in the *Not*I site of the vector pSEVA421 [17]. The pMiS1-ges-mva vector [4] contains the MVA pathway from *M. xanthus* under the control of the promoter *rhaP*_*BAD*_; transcription from this promoter can be induced by addition of *L*-rhamnose. To overproduce *P. putida* or *E. coli* enzymes involved in IPP and DMAPP synthesis, genes coding for these enzymes were cloned downstream of the IPTG-inducible promoter *Ptrc* in the vector pSEVA424 [17]. To construct the p424-DXS vector, a DNA fragment including the rbs region of the vector pET23 and the coding region of *P. putida dxs* gene was cloned between the sites *Eco*RI and *Hin*dIII of plasmid pSEVA424. Plasmid p424-nDXS was made in a similar way using a version of the *E. coli ribB* gene harboring the G108S mutation [14]. The p424-DXS plasmid was used as a backbone to clone the *idi* gene from *E. coli* (upstream of *dxs*, in the *Eco*RI site, rendering plasmid p424-IDI-DXS) or the *P. putida dxr* gene (downstream of *dxs*, between *Hin*dIII and *Spe*I, rendering p424-DXS-DXR). The *Ptrc* promoter in the vector p424-IDI-DXS was replaced with the *P. putida PkatA* promoter after PCR amplification using primers katA-PacI-F (5′-T G T T A A T T A A G A C G T T C T G T C T G C T A C) and katA-AvrII-R (5′-C T C C T A G G G C C G G C T A A T C G G) and cloning between the *Pac*I and *Avr*II sites to create plasmid pPkat-IDI-DXS. All plasmid constructs were sequenced and the plasmids transferred to *P. putida* by triparental mating using pRK600 as a donor of transfer functions.

### Quantification of lycopene accumulation

An overnight culture of the strain of interest was diluted to a final turbidity (OD_600_) of 0.05 in fresh LB medium. The indicated amount of IPTG or *L*-rhamnose was added to induce transcription from promoters *Ptrc* or *Pram* respectively. Cells were allowed to grow at 30 °C with vigorous aeration. After 24 h, cell growth was measured (OD_600_) and 5 ml aliquots were taken to lycopene extraction with acetone and quantification by measuring absorbance at 472 nm, as described at Rodríguez-Villalón et al. [38]. Three independent assays were performed. When indicated, lycopene levels were also measured by HPLC in an Agilent 1200 series HPLC system (Agilent Technologies), as previously described [39].

### RNA purification

The KTLYC(p424-IDI-DXS) strain was grown in LB medium at 30 °C in aerated flask and after 1 h the indicated IPTG concentration was added. At the start of the stationary phase (OD_600_ = 2.2), 1 ml of samples were collected, harvested by centrifugation and immediately frozen at − 70 °C. RNA was purified from cell pellet using RNeasy RNA Purification Kit (Qiagen) following the manufacturer’s instructions. Purified RNA was treated with RNase-free DNase I (Ambion), as specified by the manufacturer. RNA was quantified using a NanoDrop (Thermo Scientific) and its integrity was analyzed by agarose gel electrophoresis. The absence of DNA was confirmed by real-time PCR using primers for *rpoN*, as previously described [40].

### Real-time RT-PCR

Real-time RT-PCR assay was performed using total RNA preparations obtained from three independent cultures (three biological replicates). RNA was reverse-transcribed into cDNA using the first-strand cDNA synthesis kit (Roche) according to the manufacturer’s instructions. Real-time PCR was performed using LightCycler 480 SYBR Green I Master (Roche) and 0.3 μM of each primer on a LightCycler 480 Real-Time PCR System (Roche). RNA levels were analyzed by relative quantization as previously described [40]. The primers used are listed in Table 2.

**Table 2:** Oligonucleotides used for real-time RT-PCR

Name	DNA sequence
RpoN-Fw	TCGACCCGGAGCTGGATA
RpoN-Rv	CGGCTCGAACTGCTGGAT
Pp_katA-818 dir	ACCCATTCGACGTGACCAAG
Pp_katA-899 rv	GGGTTACGGTTGAGCTCCAG
Pp_ccoN-452 dir	AGCGCAACACCAAACACATC
Pp_ccoN-534 rv	GTTGACGATGTGCAGCAGTG
Pp_cioA-306 dir	ACTGACTGCGTTCTTCCTCG
xPp_cioA-400 rv	TCACCGTGGAGAAGAAGTGC

## Data Availability

The datasets supporting the conclusions of this article are included within the article.
